# Should robots be polite? Expectations about politeness in human–robot interaction

**DOI:** 10.3389/frobt.2023.1242127

**Published:** 2023-11-30

**Authors:** Eleonore Lumer, Hendrik Buschmeier

**Affiliations:** Digital Linguistics Lab, Bielefeld University, Bielefeld, Germany

**Keywords:** human–robot interaction, social communication strategies, politeness, user expectations, design implications

## Abstract

Interaction with artificial social agents is often designed based on models of human interaction and dialogue. While this is certainly useful for basic interaction mechanisms, it has been argued that social communication strategies and social language use, a “particularly human” ability, may not be appropriate and transferable to interaction with artificial conversational agents. In this paper, we present qualitative research exploring whether users expect artificial agents to use politeness—a fundamental mechanism of social communication—in language-based human-robot interaction. Based on semi-structured interviews, we found that humans mostly ascribe a functional, rule-based use of polite language to humanoid robots and do not expect them to apply socially motivated politeness strategies that they expect in human interaction. This study 1) provides insights for interaction design for social robots’ politeness use from a user perspective, and 2) contributes to politeness research based on the analysis of our participants’ perspectives on politeness.

## 1 Introduction

Politeness is an important phenomenon in human social interaction. It is a linguistic phenomenon that serves social functions in dialogues, but can also lead to misunderstandings (Holtgraves, 2021) because it stands in opposition to the cooperative principles underlying effective communication (Grice, 1975; Franke and Jäger, 2016).

**FIGURE 1:**
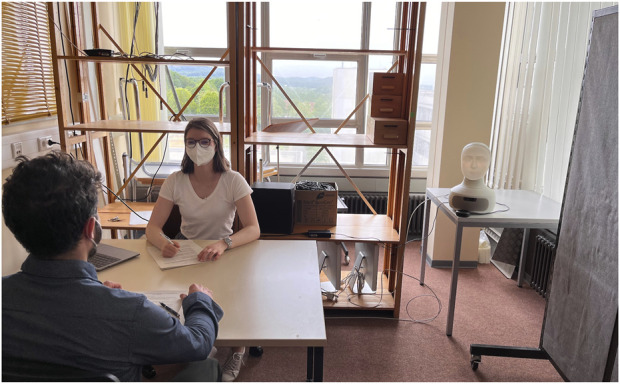
Photo illustrating the experimental setup after the Furhat robot (back right) has been revealed to the participant (front left).

Technical advances have opened up the possibility to increasingly humanize artificial conversational agents, such as embodied conversational agents or social robots. The implementation of human characteristics and language is generally considered to facilitate the interaction and improve the user experience with such agents (Gambino et al., 2020). It is questionable, however, whether it can be concluded, based on observations that humans interact similarly with robots as with other humans, that human-like interaction with agents is desirable (Hildt, 2021). This also includes the social linguistic phenomenon of politeness. Due to its complexity and the possibility of misunderstandings, it is unclear whether the implementation of social linguistic strategies is desirable and would improve the dialogues with and the user experience of robots (Clark, 2018). The study presented in this paper contributes to this debate by collecting users’ expectations about a robot’s (specifically the “Furhat” robot, see Figure 1) use of politeness.

### 1.1 Politeness

Politeness is a widely researched linguistic phenomenon relevant for tactful social interaction. Brown and Levinson (1987) introduced one of the most commonly used politeness theories, where politeness is seen as a set of strategies to save “face” (a concept originally introduced by Goffman, 1955), where face is defined as the public self-image that a person wants to preserve in interaction. Brown and Levinson (1987) formalize social influences on politeness, considering the “ranking of the imposition” by a face threatening conversational act, the “social distance” between interlocutors, and the “power” of the speaker over the hearer. They propose that speakers choose different politeness strategies based on the degree of face threat resulting from these influences. One of these strategies is indirectness, where the message of an utterance is not formulated literally but rather in such a way that it can still be interpreted as intended (Pinker, 2007). Studies found that both power and distance influence politeness in different ways (Holtgraves and Bonnefon, 2017) and also in interaction with other factors such as mood (Vergis and Terkourafi, 2015) or gender (Kasper, 1990; Holtgraves and Bonnefon, 2017).

A more recent theory of politeness that will also be discussed in our results is the “rapport management” theory by Spencer-Oatey (2008). In her theory for polite behavior she models and defines “rapport management” that is affected by the management of three aspects: “face sensitivities,” “sociality rights and obligations” and “interactional goals” (Spencer-Oatey, 2008). There are several differences between her theory and the politeness theory of Brown and Levinson (1987), but one main aspect is the different conceptualization of face and face threats. Spencer-Oatey (2008) distinguishes between behaviors that can either threaten a person’s face, sociality rights or goals, while Brown and Levinson (1987) only consider politeness strategies to counter face threats. Further, her definition of face relates face to a person’s identity, worth and dignity. Additionally, she introduces three different layers of face (individual identity, the group or collective identity and the relational identity). Parts of her theory will be discussed later on, it is, however, beyond the scope of this paper to go further into detail. Generally, politeness is a well researched phenomenon that has been analyzed in a large number of studies [see, e.g., Watts (2003); Leech (2014) for more information].

This paper focuses on politeness in human–robot interaction (HRI), specifically on users’ expectations about robots’ politeness. From the perspective of politeness research, our analysis of interview data reflects laypersons’ perspectives on politeness. Because our participants are native speakers of their language, they are proficient users of politeness in their everyday interaction. Politeness research often distinguishes between how lay people use the terms “polite” and “politeness” to talk about “their own or others” social behavior” (cf. Locher and Watts, 2005, p. 15; referred to as first order politeness or “politeness_1_”) and the theoretical view on politeness, including, for example, the above-mentioned theory by Brown and Levinson (1987), referred to as second order politeness or “politeness_2_” (Watts, 1992; Locher and Watts, 2005). Scholars have discussed this binary distinction, criticizing its simplicity by emphasizing the need for more perspectives on the complex phenomenon of politeness (House and Kádár, 2023). House and Kádár (2023), for example, combine these two perspectives in their approach. In this paper, we compare our bottom-up findings based on laypersons’ perspectives on politeness (arguably politeness_1_) with theories on politeness (politeness_2_). We thereby also contribute to politeness research by building a bridge between these two perspectives.

The relevance of politeness for HRI has already been established in numerous studies concerned with politeness in HRI in different ways (Ribino, 2023), some of which are presented in the following.

### 1.2 Politeness in human–agent interaction

Apart from the question whether social phenomena in human interaction are simply transferable to human–agent interaction (Clark, 2018), research found contradicting results for users’ perception of and expectations regarding politeness use by robots. Several studies focusing on users’ perception of how robots use politeness have found positive effects of politeness use in artificial agents, for example, regarding the perception of robots’ likability (Salem et al., 2014), persuasiveness (Hammer et al., 2016; Iop, 2022), compliance (Lee et al., 2017), and trust (Kumar et al., 2022b).

There is also a lot of research on how humans use language with machines, especially in terms of politeness (Ribino, 2023). Some studies have shown that users are polite to machines (Nass et al., 1999) and embodied conversational agents (Hoffmann et al., 2009), for example, while evaluating an agent. This is surprising because machines do not have feelings that can be hurt. These findings led to the influential theory that humans “mindlessly” apply social strategies when interacting with artificial agents [CASA, Nass and Moon (2000); Reeves and Nass (1996); Gambino et al. (2020)]. More recent studies have observed the use of politeness in the form of indirect speech acts towards robots (Williams et al., 2018), also comparing different cultural backgrounds (Seok et al., 2022). In addition to these research findings, the use of polite language towards artificial agents is also being discussed by users themselves and society at large. This, for example, has led to demands and eventually resulted in an implementation of a feature (called “magic word”) that can be enabled to require children to show politeness-based manners (saying “please” and “thank you”) when interacting with Amazon’s voice assistant “Alexa” (Elgan, 2018).

In this study, however, we focus on the expectations that potential users have before interacting with a robot. In seeming contradiction to the above studies, previous research that has explored the expectations people have when interacting with artificial agents has found that social behavior on the part of these agents is not expected and is often considered inappropriate (Clark, 2018; Clark et al., 2021). Eliciting expectations about robots is relevant because it provides further insights for dialog and interaction design (Edwards et al., 2019; Marge et al., 2022).

In a qualitative interview study, in which no robot was present, Clark et al. (2019) found that participants considered potential communication with agents to be mostly task-oriented and discussed an asymmetry in the human–robot relation and a lack of interest in building a relationship. They also argue that communication with agents is a new genre of interaction. This lack of attribution of social functions to artificial agents in an abstract discussion stands in opposition to the observations of humans’ use of politeness in interactions with robots described above (Nass and Moon, 2000; Gambino et al., 2020) and the positive perception of politeness use by agents in interaction (Inbar and Meyer, 2019; Kumar et al., 2022b).

The appearance of robots has been found to influence user perception and evaluation. For example, Rosenthal-von der Pütten and Krämer (2015) has suggested that the degree of human-likeness should be aligned with the actual capabilities of the robot. The authors argued that limiting humanoid features and aligning them with typical human abilities can reduce the negative feelings towards humanoid agents commonly described as the uncanny valley effect (Mori, 1970/2012). This is also in line with the idea of the habitability gap, which results from a mismatch between the expectations and the actual capabilities of an agent (Moore, 2017). In addition to the appearance of robots, a more human-like voice also resulted in a higher acceptance of more human-like language use, including indirect and polite language (Clark et al., 2021).

It is therefore important to consider or control aspects—such as an agent’s appearance—when querying potential users’ expectations regarding robots. Otherwise purely abstract ideas might diverge from the actual interaction experiences, resulting in the mentioned contradiction.

In this paper, we present a semi-structured interview study, collecting expectations regarding politeness in human–human compared to human–robot language-based interaction. Participants discussed their general expectations regarding politeness in human-human interaction (HHI) and, in a second phase, alleviating the mentioned contradiction, participants were confronted with a Furhat robot while talking about their expectations regarding politeness in human–robot dialogue.

### 1.3 Hypotheses

Our main research questions concern similarities and differences in expectations of politeness in dialogue as well as factors influencing human–human and human–robot interaction. Due to the exploratory nature of our qualitative interview approach we formulated two general hypotheses. First, based on a theoretical definition of politeness (Brown and Levinson, 1987) we hypothesize that politeness is seen as being used to avoid face threats.

H-1: Politeness strategies derive from face threat.

Second, based on the frequently held functional view of artificial agents (Edwards and Edwards, 2021), we have the following hypothesis:

H-2: More direct speaker strategies are expected to be used by robots in comparison to humans.

By collecting users’ expectations of robots’ social language behavior before interaction, with a qualitative interview approach, this study contributes to improving user experience (UX) design for social robots (Lindblom et al., 2020). Additionally, this study provides insights into lay perspectives on politeness thereby also contributing to politeness research.

Overall, our results show clear differences in users’ expectations regarding the robot’s use of politeness compared to humans’ use of politeness. Analyzing lay peoples’ views on politeness we found two types of politeness strategies. Based on these, we discuss implications for dialogue design for social robots.

## 2 Materials and methods

To collect participants’ expectations regarding politeness use in HRI compared to HHI, we conducted semi-structured interviews that we analyzed with a thematic analysis. In the following we will describe our method by describing the study procedure, our participants as well as the data analysis.

### 2.1 Procedure

The data collection consisted of audio-recorded semi-structured interviews (Adams, 2015; Bethel et al., 2020) conducted in German in June and July 2022 with approval of Bielefeld University’s ethics review committee (reference no. 2022-084). The interview guide (translation is available in Supplementary Section S2 of the Supplementary Material) was additionally checked by an independent researcher with expertise in conducting semi-structured interviews.

Each interview was structured according to five main topics: general understanding and perception of politeness, influences on politeness, general perception and attitude towards robots, expectations regarding politeness use by robots compared to humans and a short re-evaluation of changes in expectations after short interaction. The interviews started with questions about politeness in general and influences on politeness. For this part the interviewer did not specify whether the discussion was about human-human or human–agent interaction and all participants referred to inter-human interaction. After this first part, the Furhat robot (Furhat Robotics, Stockholm, Sweden) was revealed. Participants were then asked about their general impression of the robot followed by their expectations regarding the robot’s communication and use of politeness—in general as well as in comparison to humans and voice assistants. In order not to influence participants’ expectations the interviewer did not provide any specific information about the robot at this stage during the interview. Further, participants were also not aware that they would have the opportunity to interact with the robot at the end of the interview. Based on Lumer and Buschmeier (2022), who found influences of different space and roles of robots on their perceived relation to users, we included three different spatial scenarios, to further elicit discussions on the influences of location based role differences (at home, at work, in public) on politeness. The experimental setup (with the robot revealed) is shown in Figure 1.

### 2.2 Participants

Seventeen German native speakers (9 female, 7 male, 1 non-binary), most of them students (76.5%) with a mean age of 29 years (SD = 9.2) were recruited at Bielefeld University and offered a compensation of 10 EUR per hour.

Demographic data as well as participants’ technical affinity, technical interest, and previous experience with robots and voice assistants was collected using a questionnaire at the end of the study. Most participants reported a high (35.3%) or average (41.2%) technical affinity, three reported their technical affinity to be low. Similarly, most participants reported a very high (6%), high (64.7%) or average (23.5%) technical interest, and three reported their technical interest to be low. Almost half of the participants (47%) reported to have previous experience with robots and more than three quarters (76.5%) with voice assistants. See Supplementary Table S1 in the Supplementary Material for an overview of participant details.

### 2.3 Data analysis

An initial transcript of the interview recordings was generated by using automatic speech recognition (via BAS web services Kisler et al., 2017). It was then manually edited and corrected by two researchers to produce the final version used for the analysis. The interview data was analyzed qualitatively using “Thematic Analysis” (Braun and Clarke, 2006), a method based on the identification of patterns, so-called “themes,” occurring in the data. Thematic analysis is an iterative approach, where interview data is annotated using “codes” that are developed based on the content. In a later step, codes are grouped and iteratively analyzed to find themes. This is a tried and tested evaluation method for qualitative research in human–robot and more generally human–computer interaction research (Bethel et al., 2020). A similar approach was also used by Clark et al. (2019).

In our analysis, we created codes “inductively,” that is bottom-up and based on the data and not on prior research or theoretical insights. For the iterative theme formation, on the other hand, we additionally used “deduction,” also considering previous literature such as Brown and Levinson (1987).

Initial coding and iterative theme formation was carried out by the first author using the software MAXQDA (VERBI Software, 2019). The developed themes and codes were discussed and adapted with the second author. Subsequently, a research assistant analyzed and re-evaluated the already existing codes and formed an additional code system. Based on his own coding system the same research assistant formed and proposed own themes. To resolve small differences between the two theme and sub-theme structures, the two versions were discussed and merged to form the themes presented in the this article, grouped under three central topics [informed by the interview guide, cf. Clark et al. (2019)].

## 3 Results

In the following section, we present the results of the thematic analysis of the interview data. The themes are presented with their sub-themes and provided with a representative quote from the interview (translated to English and slightly edited, interview number and position in the interview are provided; the original quotes in German can be found in Supplementary Section S1.2 of the Supplementary Material). As mentioned above, the results for HHI and HRI are grouped around three topics: 1) the motivation for using politeness, 2) influences on politeness, and 3) the expectations regarding politeness strategies mentioned by participants. Section 3.1 will outline the results for human-human (HHI) and Section 3.2 for human–robot (HRI) interaction. Themes are set in small caps font, main themes in boldface and sub-themes in regular small caps. A visual overview of all the results in the form of thematic maps can be found for each topic below and in Supplementary Section S1.3 of the Supplementary Material.

### 3.1 Politeness in human-human interaction

#### 3.1.1 Motivation

**FIGURE 2:**
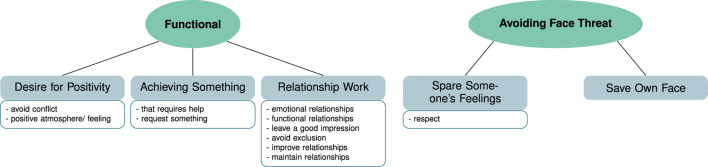
Thematic map containing the themes (green ellipses) and sub-themes (gray rectangles) resulting from the data for the topic of “motivation for using politeness” in HHI. The boxes below the sub-themes list code examples grouped to form the sub-themes.

For human–human interaction two main themes emerged describing the motivation for using politeness in conversations and situations. Some of which are also present in previous literature (Brown and Levinson, 1987; Spencer-Oatey, 2008). A visual representation of the two themes and sub-themes in form of a thematic map can be found in Figure 2.

##### 3.1.1.1 **Functional**

For this theme we grouped the codes into sub-themes where participants (*N* = 14) mentioned a functional use of politeness, that is using politeness to achieve something. This is exemplified in the following quote.

(1) *someone wants to achieve something with it [politeness], directly wants to draw a benefit from it*. (Int. 16, pos. 34)

The sub-themes that we found to form the mentioned aspects were a desire for positivity, achieving something that requires help, and building or maintaining a relationship. All aspects mentioned in the different sub-themes have in common that they comment on politeness being used to get or achieve something. This goal orientation seems comparable to the interactional goals that are part of the basis of rapport management in Spencer-Oatey (2008)’s framework. A more fine-grained analysis of the sub-themes representing the aforementioned aspects for which politeness can be used is beyond the scope of this paper.

##### 3.1.1.2 **Avoiding face threat**

Some of the aspects mentioned in our data as a reason for using politeness can be connected to the notion of face threats, similar to the theory by Brown and Levinson (1987) and Brown (2015) or the face concept by Spencer-Oatey (2008). Five participants mentioned that they or others would use politeness in order to spare someone’s feelings. This notion was also often mentioned together with the need and wish to show respect (*N* = 10). This can also be seen in the following quote.

(2) *Politeness, is showing the proper respect to […] whom you interact [with] and not putting them in an embarrassing situation*. (Int. 7, pos. 6)

These aspects can be seen as describing a form of face saving actions for the interlocutor (Brown and Levinson, 1987). In contrast, saving the face of the speaker was only mentioned by one participant as a way to use politeness “*to preserve the self-image*” (Int. 1, pos. 28).

The aspect of not wanting to hurt someone’s feelings was mentioned in relation to being empathic and aware of the other (*N* = 8).

(3) *Another side of politeness […is] in the broadest sense a benevolent attitude towards other persons and the resulting effort to preserve certain boundaries of the counterpart, in order to not bring someone into situations where he or she loses face*. (Int. 10, pos. 18)

#### 3.1.2 Influences

**FIGURE 3:**
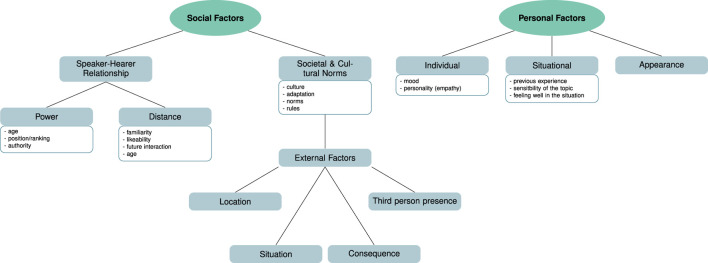
Thematic map of the main themes (green ellipses) and subthemes (gray rectangles) found for the topic of “influences on politeness” mentioned by participants in HHI. The boxes below the sub-themes list code examples grouped to from the sub-themes.

The influences on politeness strategy choice found in the data form two overall themes: the **influence of social factors** and **personal factors**. Each of these comprise several sub-themes. Figure 3 depicts these themes and sub-themes in the thematic map for this topic.

##### 3.1.2.1 **Social factors**

For this theme we grouped all participants’ statements that related to social aspects. These influences more generally arise because individuals are part of a social environment. These aspects include influences such as the speaker– hearer relationship, societal and cultural norms, and external factors—which form our sub-themes. Regarding speaker-hearer relationships, participants mentioned aspects that can be seen as related to power and distance, roughly in the sense of Spencer-Oatey (2008). This included aspects such as familiarity, likeability and the possibility of future interactions that can be grouped together to the influence of distance. Which are also in line with previous research [discussed in Spencer-Oatey (2008), p. 36]. While for aspects of power influences such as the age, status, authority of a person were mentioned. Our participants also mentioned differences in use of politeness based on the location (*N* = 7) and situation (*N* = 12)—both in line with previous research and theories (Brown and Levinson, 1987; Leichty and Applegate, 1991; Vergis and Terkourafi, 2015), which we grouped in a sub-theme as external factors that was related to the next sub-theme of societal and cultural norms. Further, the consequence of an action as well as the presence of other people was also mentioned in relation to the two aspects of situation and location.

(4) *I formulate things differently. So with friends I would just rather always be more casual […]. There [to my colleagues and lecturers] I also say what I think but I adapt that a bit to the situation.* (Int. 9, pos. 40)

The third sub-theme, societal and cultural norms, more generally includes aspects such as cultural influence that were discussed to result in adaptation and an adherence to norms and rules that are not explicitly written down but learned during childhood.

(5) *when you are greeted, that you greet back and simply a few rules of conduct that actually everyone knows without having them explicitly written down* (Int. 9, pos. 10)

##### 3.1.2.2 **Personal factors**

As already mentioned, in addition to external social influences, the data shows clear influences of personal aspects, which can be divided into the sub-themes individual influences, situational perspective, and appearance. For these influences participants mentioned aspects such as the mood of a listener or speaker to influence the politeness choice or the personality of a person (aspects of individual influences) or the previous experience or feeling well in a situation (as part of situational perspectives).

(6) *But of course also with the situation in which I am at the moment, that is, the mood, form of the day and so on. But also with the external situation of my counterpart. What’s going on in his life right now, is he somehow sad, happy and so on. It depends on a situational intuition.* (Int. 10, pos. 42)

#### 3.1.3 Expectations about politeness strategies

**FIGURE 4:**
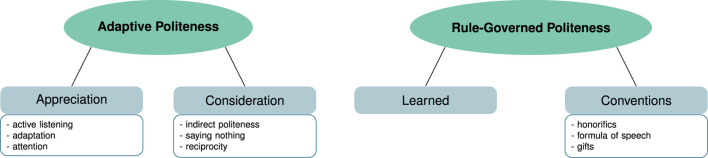
Thematic map of the main themes (green ellipses) and sub-themes (gray rectangles) for the topic “expectations about politeness strategies in human-human interaction.” The boxes below the sub-themes list code examples grouped to from the sub-themes.

Based on the motivations and influences, participants expected and talked about using different politeness strategies and forms. Our data shows two opposing forms of politeness, namely, **adaptive politeness** and **rule-governed politeness** which form the themes for this topic. A graphical representation of these two themes with sub-themes and examples from the codes are shown in the thematic map in Figure 4.

##### 3.1.3.1 **Adaptive politeness**

Resulting from the personal factors influencing politeness and the motivation to save face outlined above, participants mentioned politeness strategies oriented towards the individual listener (*N* = 14). Participants mentioned that a form of politeness was showing consideration by adapting to and appreciating the interlocutor. These included active listening or showing attention in some way (*N* = 5) and adapting to the interlocutor (*N* = 5) to show appreciation as well as using indirectness (*N* = 14), for example, in the form of white lies, or using reciprocity (*N* = 9), for example, by being considerate. One instance of this is shown in the following quote.

(7) *I think that politeness has a lot to do with the attention you pay to your interaction partner […] active listening, for example, is also a sign of politeness, that is, listening to someone and responding to what someone says.* (Int. 6, pos. 9)

Due to the focus on the individual in politeness strategies, this form of politeness, involving the strategies just mentioned, is in line with face-based theories of politeness, such as the one by Brown and Levinson (1987).

##### 3.1.3.2 **Rule-governed politeness**

On the other hand, societal influences, especially cultural and social norms were often mentioned as leading to a form of rule- and convention-based politeness (*N* = 7). In this case, politeness was seen as learned and based on conventions, such as formula of speech like saying “thank you” or using honorifics (e.g., formal addresses in German using *Sie* instead of *du*). This is illustrated in the following quote.

(8) *something like saying please and thank you, is I think part of politeness and also the communication, so the way to address someone* (Int. 1, pos. 6)

This form of politeness is more in line with viewing politeness as an expression of discernment, where politeness is seen as the result of social dynamics rather than the result of personal strategic choices (Ide, 1989; Gretenkort and Tylén, 2021).

### 3.2 Politeness in HRI

#### 3.2.1 Motivation

**FIGURE 5:**
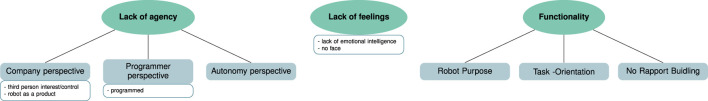
Thematic map for the topic of “motivation for politeness use” for robots. The map contains the main themes (green ellipses) and sub-themes (gray rectangles). The boxes below the (sub-)themes list code examples grouped to form the (sub-)themes.

Grouping participants’ statements on possible motivations for robots to use politeness (or not) resulted in three main themes: robots’ **lack of agency** , their **functionality** , and their **lack of feelings** . Together with their sub-themes a graphical representation of these themes is provided in Figure 5.

##### 3.2.1.1 **Lack of agency**

This theme concerns aspects where participants (*N* = 16) mentioned the control of a robot’s general behavior, including its use of politeness, being influenced by other parties. It was therefore seen as lacking agency and thus the ability to actually be polite by itself:

(9) *So yes, I’m just wondering if politeness is a purely human ability […] because a robot is, for me, well, it’s not polite for me, maybe the people who […] programmed it. That is, whether they paid attention to whether the robot is polite or not.* (Int. 11, pos. 138)

The entities actually controlling the robot’s behavior that participants mentioned were programmers and companies, as exemplified in the following quote.

(10) *It [the robot] is a product that is being sold. And it would surprise me if the people who have programmed it would build in that it should be dishonest.* (Int. 13, pos. 172)

From the perspective of the company, the robot was seen as a product. Participants therefore expected the robot’s politeness behavior to reflect companies’ interests. The robot was also seen as being programmed and therefore also being controlled by a programmer. This is in line with previous research that has also considered the relevance of third party involvement for politeness aspects in HRI (Clark, 2018).

##### 3.2.1.2 **Lack of feelings**

Related to the lack of agency and control by humans, the robot was often (*N* = 13) described as lacking feelings and empathy:

(11) *I would not expect the robot to be annoyed and therefore impolite, because it is a robot, that […] executes its program. It does not somehow have these emotions.* (Int. 2, pos. 108)

Related to the lack of feelings, the lack of face [again broadly in the sense of Brown and Levinson (1987)], also influences a robot’s expected use of politeness. One participant mentioned the lack of face together with the possibility of programming it to simulate having face:

(12) *Robots can also be programmed in such a way that they somehow pretend to have this need [to save its own face] […] They don’t have it, but I can imagine that someone, just to see how others react to it, could program it in such a way that it is able to act as if it were offended.* (Int. 10, pos. 360)

##### 3.2.1.3 **Functionality**

Most participants (*N* = 16) described the use of politeness or the lack of it by a robot to be rooted in its functionality, task-orientation and purpose. Based on its purpose the robot was expected to be programmed to have a certain politeness strategy, which aimed at fulfilling a specific task (task-orientation). This task-motivated view of politeness can be observed in the two following quotes.

(13) *In the train station its primary function is really to be nice. To show people, look, we have a good service system.* (Int. 13, pos. 150)
(14) *You ask a question and get an answer, so there’s no unfriendliness or friendliness in there. I wouldn’t assign that to a robot anyway, that it would manage that. It is all made by humans. […] Of course it has something to do with politeness, but it is made by humans.* (Int. 14, pos. 80)

#### 3.2.2 Influences

**FIGURE 6:**
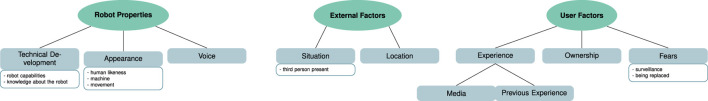
Thematic map for the topic of “influences on politeness” in HRI containing the main themes (green ellipses) and sub-themes (gray rectangles). The boxes below the sub-themes list code examples.

For HRI, motivation and influences on politeness seem to be even more interrelated than for in human-human interaction. Overall, our data revealed three categories of influences forming three main themes: **robot properties** , **external factors** and **user factors** . Again a graphical overview of themes and sub-themes can be found in the thematic map in Figure 6.

##### 3.2.2.1 **Robot Properties**

This theme contains all data that mentions different aspects of the robot as an influence on its us of politeness. This was sub-categorized into different aspects concerning the technical development and the appearance of the robot, forming our two sub-themes. Participants (*N* = 11) regarded the technical development of the robot to be relevant for the possibility of politeness behavior, including the capabilities and the knowledge that users have of the robot. In this regard participants mentioned the different expectations between the state-of-the-art and possible future developments, as evidence by the following quote:

(15) *I personally cannot yet imagine to really have a deep conversation with a robot, but maybe the technology and artificial intelligence will do that in a few years—who knows.* (Int. 16, pos. 140)

Further, the appearance of the robot was often mentioned (*N* = 12) with regard to expectations of politeness. This especially concerned humanoid features of robots that participants preferred for social abilities, such as the use of politeness, as discussed in the following quote. This preference of alignment of humanoid features and abilities is in line with previous findings (Rosenthal-von der Pütten and Krämer, 2015) and discussions (Moore, 2017; Clark et al., 2021).

(16) *depending on what the person wants […] either [a robot] with a face, which is then actually just like the other but you have the feeling he is not. Or just a squared box if you just want rational answers. […] that would just be less people-oriented and would not ask questions […]* (Int. 14, pos. 404)

##### 3.2.2.2 **External factors**

Participants were explicitly asked about their expectations of the robot’s behavior in three situations in different spaces: at home (private space), and in a work space, or in a public space. Most participants believed there to be an influence of space (*N* = 10) on the robot’s politeness behavior (location), and only a few (*N* = 4) did not expect any differences. Those who expected differences between spaces, discussed that, in a public or work setting, the robot would be task-oriented and specialized. This was in contrast to an adaptive and customizable politeness expected in a private setting that will be discussed in Section 3.2.3. Further, a few participants (*N* = 5) also believed that the robot could adapt its politeness strategy based on the situation.

(17) *I would expect a robot like that [at home] to have a greater ability to react to different things, not just [like] a train station robot that only understands things related to the train or the office robot that only understands things related to the office. It should also have a basic capacity of emotional intelligence and the ability to react appropriately to the mood, perhaps to the tone of voice in which you talk to it. And yes, definitely being human-like. So if it is used at home, then one can calibrate it to a person and supply it with more information, so that it is able to react more appropriately to statements and collect further information about this person.* (Int. 7, pos. 94)

##### 3.2.2.3 **User factors**

A further influence on the expectations regarding robot’s use of politeness in our data were aspects concerning the user. We found influences of experiences (*N* = 5) of the user, possible fears (concerning surveillance and the fear of being replaced, *N* = 5) and the ownership of the robot (*N* = 4). This last aspect can, for example, be seen in the following quote.

(18) *If he [the robot] is in my private space […] it can do that [defined tasks] neutrally. Because I bought it for this task, then I don’t need any special politeness.* (Int. 2, pos. 136)

In addition to previous experience with artificial agents, the sub-theme of experience also contained the influence of the media on the expectations participants had regarding the robot’s politeness. In line with previous research (Rosenthal-von der Pütten and Krämer, 2015), some participants (*N* = 3) mentioned movies when formulating their expectations of the robot.

(19) *Through science fiction, you are somehow already trained and have expectations.* (Int. 5, pos. 215)

#### 3.2.3 Expectations about politeness strategies

**FIGURE 7:**
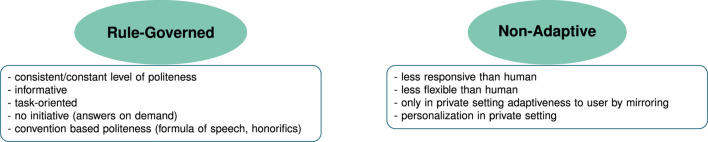
The thematic map shows the two main themes (green ellipses) for participants’ “expectations regarding politeness” in HRI. The boxes below the themes list code examples grouped to from the themes.

With the background of the influences and motivations mentioned so far, two main themes were found describing the expectations participants had regarding the robot’s use of politeness: **Rule-Governed** and **Non-Adaptive** . Figure 7 shows these two themes in a thematic map alongside the most common codes.

##### 3.2.3.1 **Rule-governed**

Participants often described expecting robot’s use of politeness to be similar to learned and rule-based politeness as applied by humans (see Section 3.1.3). This rule-based politeness behavior of robots therefore included the use of standardized phrases, sets of expressions that were learned and trained (*N* = 8). This can be seen in the following quote.

(20) *I expect politeness coming from social rules, because the robot has no empathy. And if it does, then it has the empathy that was programmed into it, and that’s why I don’t expect it to empathize with my position, so to speak, but the developers have to do that beforehand. So I expect the robot to be polite, because that’s what it learned.* (Int. 1, pos. 113)

This kind of politeness was described as resulting from the previously mentioned task-orientation and functionality of the robot especially focusing on informativeness and neutrality (*N* = 14). Further, due to the lack of feelings and social abilities of the robot (*N* = 11), politeness was expected to be constant (*N* = 8), as also described in the following quote.

(21) *because I mean those are just humans who are sometimes annoyed or hungry or have a bad day. And it [the robot] doesn’t have all that. So he would always be polite at a constant level.* (Int. 1, pos. 80)

Some participants (*N* = 6), however, expected rule-based politeness to be face-saving to a certain point, such that the robot was expected to be dishonest if brought in a situation where it could hurt the feelings of the user, as described in the following quote.

(22) *These are still human-made machines. I think people tend to not like it when you’re honest, that is, when you’re really honest with them […] it sometimes makes people feel uncomfortable. That’s why I think that robots also tend to be less honest.* (Int. 9, pos. 120)

##### 3.2.3.2 **Non-adaptive**

The second theme is complementary to the rule-based expectations of politeness. In this theme we grouped aspects for which participants mentioned expecting a lack of flexibility, in the sense that the robot is not expected to adapt to the user or the situation. This is exemplified in the following quote.

(23) *I think that it is programmed to be polite in order to not say anything mean. But I don’t think it can react to situations like a human being. And therefore it can probably sometimes seem rude unintentionally. […]* (Int. 12, pos. 74)

Further, the robot was described to be less responsive than a human as it was not expected to have the same possibilities to react using facial expressions, for example,. It was also seen as only reactive and not taking initiative and not being able to adapt to the user.

The only adaptation participants expected of the robot was in a private setting. Here participants (*N* = 9) expected the possibility to personalize or customize the robot to the desired politeness strategy.

(24) *I think that at home there is the possibility to adjust that you talk to each other in a more relaxed tone.* (Int. 13, pos. 156)

The adaptation that was expected beyond customization was mirroring the user’s politeness behavior (*N* = 2), as discussed in the following quote.

(25) *I would expect that it is incredibly polite in the initial period, but that it is set to “mirror me”, to understand my way of communicating, to process and to include it in its way of communicating, that it drops this extreme politeness, because I expect someone who is in my home permanently or regularly, not to be someone who is incredibly polite to me.* (Int. 8, pos. 110)

## 4 Discussion

In the following section we will summarize and discuss the results presented above, beginning with a discussion of the differences between HRI and HHI with respect to the perception of politeness. We will then discuss the differences between HRI and HHI concerning the expectations regarding politeness. Finally, we will discuss possible implications for the design of human–robot interactions.

### 4.1 Politeness influences and perception

When analyzing participants’ views, we found that politeness is often perceived as being used to show respect or to avoid hurting someone’s feelings [e.g., shown in quotes (2), (3)]. These aspects can be considered as face concerns, that are comparable to theoretical face concepts by Brown and Levinson (1987) and Brown (2015) and more so by Spencer-Oatey (2008). This leads us to accept our first hypothesis that even from a lay perspective politeness is often used due to face threat. This type of politeness concerning face influenced by the speaker–hearer relationship [e.g., shown in quotes (4)] and personal factors resulted in politeness strategies that we defined as being adaptive to the interlocutor. Influences mentioned by participants such as the mood or speaker–hearer relationship are in line with previous research on politeness (Brown and Levinson, 1987; Vergis and Terkourafi, 2015). These aspects of politeness were, however, only mentioned in the context of HHI.

Participants also mentioned politeness to be used to achieve something, in which case the choice of politeness was described as being influenced by societal and cultural norms. These aspects resulted in strategies that we defined as rule-governed politeness strategies. This view of politeness resulting from social dynamics without the influence of personal strategic choices is in line with the definition of politeness as discernment (Ide, 1989; Gretenkort and Tylén, 2021).

Participants’ views present in our data suggest that the two perspectives on politeness known in the literature—face based politeness (Brown and Levinson, 1987) and politeness as discernment (Ide, 1989)—could be regarded as complementing each other and not necessarily contradicting each other, as also discussed in Gretenkort and Tylén (2021).

Furthermore, the socially and culturally grounded rules leading to politeness strategies that we called rule-governed based in our data, can also be compared to Spencer-Oatey (2008)’s “sociality rights and obligations” in interaction with “interactional goals”. As shown in the results of our data (Section 3), it seems that these two bases for rapport management (politeness) by Spencer-Oatey (2008) are not separated. In our data, the functional aspects (similar to “conversational goals”) underlie the politeness strategy choices, with adaptive politeness being similar to Spencer-Oatey (2008)’s face concept and rule-governed politeness strategies, arguably, being comparable to her “sociality rights and obligations.”

The rule-governed form of politeness was also present in participants’ discussions of politeness in HRI. The parallels we found with the forms of politeness mentioned for HHI were the task-orientation, i.e., the functional aspect of robots’ use of politeness [e.g., in quotes (13), (14)], which in part can be seen as similar to the functional aspects of politeness in HHI [e.g., in quote (1)]. The lack of feelings and agency of robots [e.g., in quotes (11), (9)] together with technical restrictions and their (humanoid) appearance [e.g., in quotes (15), (16)] lead participants to expect rule-governed politeness [e.g., quote (20)], which will be described in Section 4.2. A further aspect that lead participants to their expectations were their previous experiences, the media influence and the fear of being replaced or monitored, which are also aspects discussed in the HRI literature especially in the context of the uncanny valley effect (Rosenthal-von der Pütten and Krämer, 2015).

To control for the influence of a brief interaction on prior expectations (as seen in Edwards et al., 2019), a number of participants (*N* = 15) interacted with the Furhat robot after the interview^1^. The answers to whether their prior expectations had changed after the interaction differed widely. Some of the participants did not answer clearly to the question (*N* = 4). Of the rest, half of the participants (*N* = 6) claimed to have the same expectations as before the interaction, while the other half (*N* = 5) claimed to have changed their views on politeness for robots. The participants who claimed to have the same expectations as before the interaction seem to have higher technical interest and affinity on average than those who changed their expectations after interaction (see Supplementary Table S1 in our Supplementary Material). Further, we observed, that before the interaction some participants (*N* = 2) had overall low expectations of the robot, based on the robot’s appearance and lack of movement. As the methodology had some minor issues (the interaction was in English and participants were asked directly), these post-interaction comments can only be taken as an indication of possible changes in participants’ views on politeness after the interaction (Edwards et al., 2019). Even though these insights are in line with previous research regarding the influence of prior experience and technical affinity on expectations (Luger and Sellen, 2016), future research should replicate these findings with more participants.

As the scope of this paper was to investigate user expectations before an interaction, we will discuss below the two types of politeness strategies that were present in our data.

### 4.2 Politeness expectations

As mentioned above, for HHI, we identified two types of politeness strategies in our data: adaptive politeness and rule-governed politeness.

Adaptive politeness are strategies that are used to show appreciation and consideration. They include active listening, demonstrating attentiveness, reciprocity, indirectness and adapting to the listener. These politeness strategies are consistent with face-oriented politeness strategies as mentioned by Brown and Levinson (1987) or the face concept by Spencer-Oatey (2008). Most participants did not consider these types of strategies for robots, thereby excluding the possibility that the robot could adapt to different users due to its lack of flexibility and technical limitations mentioned above. The only case where some participants expected the robot to adapt to the situation or the user was in private settings [e.g., see quote (24), (25)]. Here, participants expressed the wish to be able to customize the robot’s politeness behavior or have the robot adapt to their own language choice over time. Some aspects that participants considered to be part of adaptive politeness, are active fields of research in human-agent interaction, e.g., the ability to display active listening behaviors when humans are speaking (e.g., Gratch et al., 2007).

Rule-governed politeness is similar in both HRI and HHI. Participants mentioned politeness based on societal rules and norms, which included a fixed set of expressions that are learned. Examples are saying “thank you” or using honorifics. Additionally, the expectations of rule-governed politeness used by robots also included the expectation of a constant (that is not situation specific) task-oriented politeness that should not change the informativeness of the robot’s utterances. This confirms our second hypotheses, that participants would expect more direct language from robots in general [based on the task-oriented and functional view of robots (Clark et al., 2019; Edwards and Edwards, 2021)]. Still, further research is needed to complement these impressions.

We believe that considering the two types of politeness strategies found in our data is also useful for future research on the acceptance of robots in terms of their use of social linguistic strategies. The distinction between these types of strategies allows for a more nuanced study than the analysis of a robot’s use of polite or impolite language, which is common in experimental studies (see Ribino, 2023).

Overall, linguistic phenomena are culturally influenced, therefore, as has also been observed in previous HRI studies, the perception of the use of politeness by artificial agents is culturally influenced (e.g., Kumar et al., 2022a). It is therefore important to consider the results in the cultural context (Germany) of the study. Furthermore, the age of users may also influence their expectations (e.g., Kumar et al., 2022b). Given the average age of our participants (29 years), our results should be interpreted primarily for a younger group of users. In addition, most of our participants reported being relatively interested in technology and having had previous experience with voice assistants. As seen in our results (Section 3.2.2, theme: user factors), our participants’ previous experience with artificial agents influenced their expectations regarding the politeness of the Furhat robot. A replication of the study with older participants and participants without previous experience with voice assistants would be informative to consider another potential user group. However, general design implications can be drawn from this user perspective, based on the distinction between adaptive politeness and rule-governed politeness.

### 4.3 Implications for robot interaction design

The design of human-robot interactions can be guided by the two types of politeness identified in our data. Dialogue design for social robots can be facilitated by distinguishing between rule-governed and adaptive politeness, as it helps to inform decisions about the robot’s use of social language and enables a more differentiated consideration of the topic. Interaction designers can choose to implement only basic rule-governed politeness, or make informed decisions about implementing aspects of more complex adaptive politeness strategies.

We propose the use of culturally adapted rule-governed politeness by robots as a good basis for successful interactions. We believe that a well-functioning implementation of these expected politeness strategies would contribute to the acceptance of conversational robots in public settings. Since our potential users already expect this type of politeness strategy, we believe that it could facilitate interaction by making the robot culturally adapted but task-oriented. This partly contradicts Ribino (2023)’s suggestion that machines should generally be more adaptive to the user when it comes to politeness. Our results suggest that adapting a robot’s politeness behavior to the user may only be necessary in a private setting, and not in a public one.

These basic rule-based politeness strategies have often already been implemented in robots, for example, in previous studies focusing on the “social rules of etiquette” (Ribino, 2023). Etiquette is included in our rule-governed politeness strategies, as it concerns appropriate behavior derived from social conventions (Hayes and Miller, 2011).

Another aspect that emerges from our data is the desire for personalization of the robot’s politeness behavior and for the robot to adapt to the user over time. Our data suggest that this adaptation and personalization is relevant in private settings, where a limited number of regular users are present. Adaptation in this case was considered to mirror the user’s politeness strategies gradually over time. We therefore suggest that social robots used in users’ homes should include an option to enable more complex adaptive politeness strategies. This would allow users to choose whether they want the robot to speak in a human-like social way, by showing attentiveness and considering the user’s feelings, or by mirroring the user’s own social language strategies.

Allowing the user to choose different politeness settings, such as more sophisticated adaptive politeness strategies, might, however, have ethical consequences. These might arise from the possibility that the robot might have to answer untruthfully in order to be polite and not hurt the user’s face, a politeness strategy common in human interaction, for example, in the use of white lies. This ethical issue needs to be considered in the dialogue design process when implementing adaptive politeness strategies.

Overall, however, our design suggestions need to be considered in the context of the robot and its current technical capabilities and development. As previous research has shown, there are several factors that influence the perception and expectations of robots. As discussed above, appearance is one of them. However, other aspects such as the movement (or more specifically, the behavior) of machines influence how they are perceived (Rosenthal-von der Pütten and Krämer, 2015; Clark et al., 2019). As the Furhat robot used in this study is a conversational robot head (without a body), future research could consider these aspects to replicate our findings. Overall, our data, however, suggest the alignment of task relevance, functionality, technical capabilities, and appearance (especially regarding humanoid features), as has been discussed in previous studies and literature (Rosenthal-von der Pütten and Krämer, 2015; Moore, 2017; Clark et al., 2019).

To further validate our results, we plan to implement the two proposed sets of politeness strategies in a Furhat robot and investigate the robot’s acceptance and participants’ perceptions of the strategies. Furthermore, this study should be replicated to ask participants about their expectations after interacting with a robot, in order to control for the effect of an actual interaction on expectations of politeness. In addition, we suggest replicating this study with a different type of conversational agent, including non-embodied agents such as Amazon’s Alexa voice assistant.

## 5 Conclusion

This paper presents a semi-structured interview study on users’ expectations of robots’ use of politeness compared to humans, analyzed using thematic analysis, a qualitative research method. The data reveal two types of politeness strategies in human interactions. On the one hand, participants considered *rule-governed politeness* strategies, which arise from social and cultural norms and include the use of a fixed set of expressions and honorifics. We compared these strategies to the combination of the notions of sociality rights and obligation and interactional goals Spencer-Oatey (2008). On the other hand, they considered *adaptive politeness* strategies, which result from social and personal considerations and lead to more complex use of politeness, for example, being indirect by telling white lies, showing appreciation through active listening, or by adapting to the listener. We consider this latter form of politeness that participants’ describe, to be similar to the face-based politeness theories of Brown and Levinson (1987) or the face concept by Spencer-Oatey (2008).

By comparing the two politeness strategy types, that were found bottom-up in this study, with already existing politeness research, we are connecting the two perspectives mentioned in politeness research, namely, politeness_1/2_ (Watts, 1992; Locher and Watts, 2005). The presented approach shows, that lay people’s intuitive conceptions of politeness (politeness_1_) are a valuable addition to theories and align in certain aspects with theoretical frameworks (politeness_2_). Similar to other scholars (e.g., House and Kádár, 2023), we therefore argue that this strict binary distinction might limit politeness research, as it would exclude the valuable insights that can be gained by combining the two perspectives as in the current study. In future research, we would like to focus more on the theoretical insights from these data (this would be beyond the scope of the current paper, which focuses on insights for HRI research).

Applied to human–robot interaction, our data shows that users only expect humanoid conversational robots to use rule-governed politeness strategies (at least before they interacted with a robot).

Involving potential users prior to the actual design process is important in order to improve the development of the user experience in human–robot interaction (Lindblom et al., 2020). Design implications are therefore discussed based on the distinction found between rule-governed and adaptive politeness strategies. We suggest that basic rule-governed politeness, adapted to the culture in which the robot is used, is fundamental to the acceptance of language-based human–robot interaction. Furthermore, our data suggests that users of social robots at home might want to personalize the politeness strategies and social behavior of their social robot. We therefore believe that in home settings, dialogue design should be adaptive to users and potentially include aspects of adaptive politeness strategies.

## Data Availability

The raw data supporting the conclusion of this article will be made available by the authors, without undue reservation.

## Footnotes

1 Using the English language demo “Meet Furhat” from the Furhat skill library, see https://youtu.be/v9ZNHDYPjo8
