# Supplementary material for: Should robots be polite? Expectations about politeness in human–robot interaction
Source: Front Robot AI. 2023 Nov 30;10:1242127. doi: 10.3389/frobt.2023.1242127 (PMC10720661; doi:10.3389/frobt.2023.1242127)
Supplement: Supplementary file 1 [file DataSheet1.PDF]

# Supplementary Material

## 1 SUPPLEMENTARY DATA

### 1.1 Demographic data

**Table S1.** Participant's demographic data and answers to questions regarding their self assessed technical affinity and interest as well as previous interactions with robots and voice assistants. Where P are the participant numbers that correspond to the interview number used for the quotes of the data. Additionally, the last column contains the information whether participants changed their expectations after the interaction with the robot (see section 4.1 of the paper).

| P | Gender | Age | Highest Degree | Technical Affinity | Technical Interest | Previous Interaction with: Robots | Previous Interaction with: Voice Assistants | Change After Interaction |
|---|---|---|---|---|---|---|---|---|
| 1 | f | 26 | Master | High | High | No | Yes | No |
| 2 | m | 20 | A-Levels | High | Very High | Yes | Yes | Yes |
| 3 | f | 20 | A-Levels | Low | Low | No | No | No |
| 4 | f | 26 | A-Levels | Low | Average | No | Yes | No |
| 5 | m | 44 | Master | High | High | Yes | No | Yes |
| 6 | f | 28 | Master | Low | Low | No | Yes | Unclear |
| 7 | m | 24 | A-Levels | Average | Average | No | No | Yes |
| 8 | m | 25 | Apprenticeship | Average | High | Yes | Yes | No |
| 9 | f | 32 | Master | High | High | Yes | Yes | No |
| 10 | m | 36 | Bachelor | Average | High | No | No | Unclear |
| 11 | f | 26 | A-Levels | Average | Average | Yes | Yes | Unclear |
| 12 | d | 29 | Bachelor | High | High | Yes | Yes | Yes |
| 13 | f | 22 | A-Levels | Low | High | Yes | Yes | No |
| 14 | m | 26 | Bachelor | Average | Low | Yes | Yes | Unclear |
| 15 | f | 26 | A-Levels | Average | Average | No | Yes | Yes |
| 16 | m | 56 | Master-equivalent | High | High | No | Yes | No interaction |
| 17 | f | 22 | A-Levels | Average | High | No | Yes | No interaction |

## 1.2 Extended interview quotes

This section presents participants' original German quotes alongside the English and edited translation printed in the paper. For each quote, a pair of numbers (int., pos.) refers to the interview number (int.) and the position of the quote in the interview (transcript line number pos.). The combination of the interview number and position in the interview is used to reference the quote already used in the paper. The interview number and position in the transcript are provided in brackets (e.g., (Int. 8, pos. 10)). Quotes are presented in the same order as in the paper.

### Politeness in inter-human interaction

**Motivation - Directed(/ achieving a goal)**:

(1) *DE: Das Zweite ist natürlich, dass jemand irgendwo das das, dass man etwas möchte. Also weiß nicht wie würde man das formulieren? Aber dass das jemand damit etwas bewirken möchte, direkt also einen Nutzen daraus ziehen möchte joa und gut. Und das ist natürlich auch so ein bisschen abhängig davon. (EN:The second is, of course, that someone somewhere wants that, that you want something. So I don't know how you would formulate that? But that someone wants to achieve something with it, directly wants to draw a benefit from it, and so on. And that is of course also a bit dependent on it.)* (Int. 16, pos. 34)

**Motivation - Avoiding face threat**:

(2) *DE: Höflichkeit, ist den den oder die Gesprächspartner oder Leute um einen herum, mit dem man interagiert, den angemessenen Respekt entgegenzubringen und sie nicht in eine verlegene Situation zu bringen (EN:Politeness, is showing the proper respect to the person or people around you with whom you interact and not putting them in an embarrassing situation. )* (Int. 7, pos. 6)

(3) *DE: eine andere Seite der Höflichkeit, die ja die mehr, mehr irgendwie so ein mehr eine eine Einstellung ist ähm, vielleicht eine andere, im weitesten Sinne wohlwollende Einstellung gegen gegenüber anderen Personen und, ähm, dass und der der Wunsch und daraus folgendes bemühen ja, gewisse Grenzen des des Gegenübers zu wahren, also jemanden, äh, nicht in Situationen zu verbringen, wo zu verbringen, in Situationen zu bringen, wo er oder sie das Gesicht verliert. (EN:another side of politeness, which is the more, more somehow such a more an attitude is um, perhaps another, in the broadest sense benevolent attitude against towards other persons and, um, that and the desire and from it resulting effort yes, to preserve certain boundaries of the of the counterpart, so someone, uh, not to spend, where to spend, to bring into situations where he or she loses face. )* (Int. 10, pos. 18)

**Influences - Social factors**:

(4) *DE: Ich verpackt das dann anders. Also bei Freundinnen würde ich halt schon eher immer da. Ja da wird man vielleicht auch mal salopper und sagt es anders bei ehm ja, jetzt irgendwie Kolleginnen oder Dozierenden von mir selbst. Da sage ich auch schon, was ich denke. Aber da passe ich das dann eher so ein bisschen an die Situation an. (EN:I formulate it differently. So with friends I would just rather always there. Yes, maybe I am more casual and say it differently to ehm yes, now somehow colleagues or lecturers of myself. There I say also say what I think. But then I adapt that rather a bit to the situation. )* (Int. 9, pos. 40)

(5) *DE: Aber wenn man gegrüßt wird, dass man schon dann zurückgrüßt und einfach so ein paar ja Verhaltensregeln ehm die eigentlich alle kennen, ohne dass man sie explizit irgendwo aufschreibt, also nicht drängeln* *(EN:But when you are greeted, that you already then greet back and simply so a few yes rules of conduct ehm that actually everyone knows, without that you explicitly write them down somewhere, so not pushing )* (Int. 9, pos. 10)

**Influences - Personal factors**:

(6) *DE: Ehm. Aber natürlich auch mit der Situation, in der, in der ich gerade bin, also Stimmung, Tagesform und so was, aber auch mit der mit der mit der äußeren Situation meines Gegenübers. Also, was passiert bei dem gerade im Leben, ne?, ist gerade irgendwie traurig, fröhlich und so weiter. Und was braucht das Gegenüber gerade ne? und so einem situatives Gespür, von dem das abhängt.* *(EN:Ehm. But of course also with the situation in which I am at the moment, i.e. mood, form of the day and so on, but also with the external situation of my counterpart. So, what's going on in his life right now, is he somehow sad, happy and so on. And what does the other person need right now, ne? and such a situational intuition, on which that depends. )* (Int. 10, pos. 42)

**Expectations - Adaptive politeness**:

(7) *DE: ich finde ich Höflichkeit hat ganz viel mit Aufmerksamkeit zu tun, die man seinem Gegenüber schenkt. [. . . ] Und ich finde aktives Zuhören zum Beispiel ist ja auch ein Zeichen von Höflichkeit, also jemandem Gehör schenken und auf das eingehen, was jemand erzählt* *(EN:I think politeness has a lot to do with the attention you pay to your counterpart. [. . . ] And I think active listening, for example, is also a sign of politeness, i.e., listening to someone and responding to what someone says. )* (Int. 6, pos. 9)

**Expectation - Rule-governed politeness**:

(8) *DE: also so etwas wie bitte und danke sagen, finde ich gehört zur Höflichkeit dazu und auch so die Kommunikation also die Ansprache, also sieze ich jemanden oder dutze ich jemanden. Oder muss ich Doktor irgendetwas sagen oder so.* *(EN:So something like saying please and thank you, I think is part of politeness and also so the communication, so the way to address someone, so do I say you (colloquiual) way (honorifics). Or do I have to say something like doctor or something. )* (Int. 1, pos. 6)

## Politeness in human–robot interaction

**Motivation - Lack of agency**:

(9) *DE: Also ja, ich frag mich halt irgendwie ab Höflichkeit eine rein menschliche Eigenschaft ist aber inwiefern Frage ich dann, wie es der Roboter programmiert? Inwiefern haben da Menschen noch mit reingespielt, weil ein Roboter für mich ist, also ja der ist an sich für mich nicht höflich, vielleicht die Leute, die dahinter steckten und ihn programmiert haben. Das sind ob die darauf geachtet haben, ob der Roboter höflich ist oder nicht.* *(EN:So yes, I'm just kind of wondering if politeness is a purely human trait but then I ask myself how is the robot programmed? To what extent did people play a role in it, because a robot is for me, well, it's not polite for me, maybe the people who were behind it and*

*programmed it. That is whether they paid attention to whether the robot is polite or not. )* (Int. 11, pos. 138)

(10) *DE: Na ja, wenn ich gerade in so einer Situation ist es ja ein Produkt, was verkauft wird. Und da würde es mich schon wundern, wenn die Leute, die dann programmiert haben oder irgendjemand eingebaut haben, das er unehrlich zu einem sein soll.* *(EN:Well, especially in such a situation it is a product, what is being sold. And it would surprise me if the people who have programmed or someone else, would build in that he should be dishonest. )* (Int. 13, pos. 172)

**Motivation - Lack of feelings**:

(11) *DE: Ich würde aber nicht erwarten, dass ein Roboter genervt und dadurch unhöflich wird, weil es halt ein Roboter ist, dem halt nicht irgendwie ja. Der spult halt sein Programm ab. Er hat jetzt irgendwie nicht diese Emotion* *(EN:I would not expect the robot to be annoyed and therefore impolite, because it is a robot, who is not yea he executes his program. He does not somehow have these emotions. )* (Int. 2, pos. 108)

(12) *DE: na klar kann man. Kann man Roboter auch so so programmieren, dass er irgendwie so tut, als hätte er dieses Bedürfnis [sein Gesicht zu wahren] er kann ja keins haben? ne Ja, denkt also. Nein. Also das Bedürfnis hat er nicht. Aber ich könnte mir vorstellen, dass jemand ihn einfach um zu gucken, wie andere darauf reagieren, so Programm, so programmiert, dass er in der Lage ist, so zu tun, als wäre er beleidigt.* *(EN:Of course you can. Can robots be also programmed in such a way that they somehow pretend to have this need - they can't have one? ne Yes, so they think. No. So he doesn't have that need. But I could imagine that someone, just to see how others react to it, could program it in such a way that it is able to act as if it were offended. )* (Int. 10, pos. 360)

**Motivation - Functionality**:

(13) *DE: Naja, also in der Bahnhofshalle. Ist ja wirklich dann seine primäre Funktion, eigentlich nett zu sein. Den Leuten zu zeigen hier wir haben ein gutes Servicesystem. Seien Sie zufrieden mit der Deutschen Bahn.* *(EN:Well, so in the train station. Its primary function is really to be nice. To show people here we have a good service system. Be happy with the Deutsche Bahn. )* (Int. 13, pos. 150)

(14) *DE: Du stellst eine Frage und kriegst ne Antwort, also, da ist jetzt, da ist keine Unfreundlichkeit oder Freundlichkeit da drin. Also das würde ich halt einem Roboter sowieso nicht zuordnen, dass der oder die oder dass das hinkriegen würde. Es ist halt alles menschlich gemacht. [...] Also das hat natürlich schon was mit Höflichkeit zu tun, aber ist halt ja von Menschen halt gemacht.* *(EN:You ask a question and get an answer, so there's no unfriendliness or friendliness in there. I wouldn't assign that to a robot anyway, that it would manage that. It is all made by humans. [. . . ] Of course it has something to do with politeness, but it is made by humans. )* (Int. 14, pos. 80)

**Influence - Robot properties**:

(15) *DE: Und aber wie gesagt, um um um wirklich ein tiefgreifendes Gespräch zu führen, kann ich mir persönlich so noch nicht mit nem Roboter vorstellen, aber macht die Technik und die künstliche Intelligenz vielleicht mal in ein paar Jahren hergeben wer weiß* *(EN:But as I said, I cannot yet*

*imagine personally to really have a deep conversation with a robot, but maybe the technology and artificial intelligence will do that in a few years, who knows )* (Int. 16, pos. 140)

(16) *DE: Und dann würde ich halt je nachdem, was die Person möchte für sich entweder einen mit Gesicht, der dann ja eigentlich genau ist wie der andere aber man hat das Gefühl, er ist nicht so oder halt einen vier eckigen Kasten, wenn man einfach rationale Antworten möchte. So. Also, der halt weniger menschenbezogen ist und nicht Nachfragen stellt und so. (EN:And then I would just depending on what the person wants for himself either one with a face, which is then actually just like the other but you have the feeling he is not so or just a squared box if you just want rational answers. Like this. So, that is just less people-oriented and does not ask questions or anything. )* (Int. 14, pos. 404)

**Influence - External factors**:

(17) *DE: Von so einem Roboter würde ich halt, äh, ja ein höheres es eine größere Fähigkeit erwarten, auf die verschiedenen Dinge zu reagieren, also nicht bloß auf zum Beispiel ein Bahn Roboter jetzt der nur diese Bahn bezogen, bahnhofsbezogene Dinge hat oder der Kanzlei-Roboter, der jetzt nur da die Büro bezogenen Dinge versteht, sondern halt, der sollte halt auch natürlich diese ein Mindestmaß an dieser emotionalen Intelligenz aufweisen und ja, die Möglichkeit haben, angemessen darauf zu reagieren, auf die Stimmung zu reagieren, auf vielleicht auf die Tonlage, in dem man sich mit ihm unterhält. Und ja, auf jeden Fall Menschen ähnlich zu sein. Also wahrscheinlich wenn er zu Hause steht, dann kann man den vielleicht auch eine Person kalibrieren und den mit mehr Informationen versorgen, sodass der in der Lage ist, angemessener auf Aussagen zu reagieren und äh Gespräche zu führen und halt im Informationen über diese Person zu sammeln und zu verwehren. (EN:I would expect a robot like that to have a greater ability to react to different things, not just to, for example, a train robot that only understands things related to the train or the office robot that only understands things related to the office, but it should also have a minimum of emotional intelligence and the ability to react appropriately to the mood, perhaps to the tone of voice in which you talk to it. And yes, definitely being similar to people. So probably if he stands at home, then one can calibrate to a person and supply him with more information, so that he is able to react more appropriately to statements and uh to lead conversations and collect information about this person. )* (Int. 7, pos. 94)

**Influence - User factors**:

(18) *DE: Also ich denke mal so wenn man, wenn er jetzt so, weil, er wenn er bei mir im privaten Raum stehen, dann habe ich mir den angeschafft, so den hat ich mir geholt, weil ich weiß, der soll bestimmte Funktionen sein wie zum Beispiel mir sagen wenn der Kühlschrank offen, wenn ich einen Kühlschrank vergessen habe, zuzumachen. Und dann kann er das ruhig neutral machen. Weil für die Aufgabe habe ich ihn angeschafft, dann brauche ich nicht besondere Höflichkeit. (EN:So I think so if one, if he now so, because, if he is with me in the private room, then I have bought it, so I got it, because I know, he should have certain functions like for example tell me if the refrigerator is open, if I forgot to close the refrigerator. And then it can do that neutrally. Because I bought it for this task, then I don't need any special politeness.)* (Int. 2, pos. 136)

(19) *DE: Also es ist sehr fortgeschritten, das habe ich nicht anders erwartet. Und durch Science Fiction ist man ja irgendwie auch schon trainiert und hat Erwartungshaltung. (EN:Well, it's very advanced, I*

*didn't expect anything else. And through science fiction, you are somehow already trained and have expectations. )* (Int. 5, pos. 215)

**Expectations - Rule-governed**:

(20) *DE: da erwarte ich Höflichkeit aus den sozialen Regeln heraus, quasi weil der Roboter, der keine Empathie. Und wenn, dann hat er nun die Empathie, die ihm programmiert wurde und deswegen erwarte ich da kein einfühlen, quasi in meine Position, sondern das müssen halt die Entwickler machen vorher. Also da erwarte ich eher, dass der Roboter halt höflich ist, weil erst so gelernt hat. (EN:I expect politeness coming from social rules, because the robot has no empathy. And if it does, then it has the empathy that was programmed into it, and that's why I don't expect it to empathize with my position, so to speak, but the developers have to do that beforehand. So I expect the robot to be polite, because that's what it learned. )* (Int. 1, pos. 113)

(21) *DE: weil ich mein das sind halt auch Menschen, die sind mal genervt oder haben Hunger oder einen schlechten Tag. Und das hat er ja alles nicht. Also der wäre ja immer auf einem gleichbleibenden Level höflich. (EN:because I mean those are just humans who are sometimes annoyed or hungry or have a bad day. And it [the robot] doesn't have all that. So he would always be polite at a constant level. )* (Int. 1, pos. 80)

(22) *DE: Nein, weil das ja immer noch von Menschen geschaffenen Maschinen sind. Und ich glaube, Menschen tendenziell eher dazu neigen, es nicht mögen wenn man ehrlich also, wenn man wirklich ehrlich zu ihnen ist. Also man die sagen das ganz oft. Aber wenn man dann wirklich ehrlich ist, dann macht das Menschen, glaube ich doch manchmal noch so ein ungutes Gefühl. Deswegen glaube ich eher, dass Roboter eher auch dazu neigen, nicht ehrlich zu sein. (EN:No, because these are still human-made machines. And I think people tend not to like it when you're honest, that is, when you're really honest with them. So they say that quite often. But if you're really honest, I think that sometimes makes people feel uncomfortable. That's why I think robots also tend to be less honest. )* (Int. 9, pos. 120)

**Expectations - Non-adaptive**:

(23) *DE: Also ich denk schon, dass sehr wahrscheinlich so höflich programmiert ist, dass er dann keine gemeinen Sachen sagt. Aber ich denke nicht, dass es jetzt unbedingt so viele Situationen Wahrscheinlich reagieren kann wie ein Mensch. Und deswegen wahrscheinlich kann er dann manchmal unabsichtlich unhöflich wirken, so zum Beispiel irgendwas falsch verstehen. Wahrscheinlich. Oder ja, vielleicht auch dazwischenreden. (EN:So I think that he is probably programmed so polite that he doesn't say mean things. But I don't think that it can react now necessarily to so many situations as probably like a human being. And therefore probably it can then sometimes unintentionally seem rude, so for example misunderstanding something. Probably. Or yes, maybe interjecting.)* (Int. 12, pos. 74)

(24) *DE: Ja, ich denke zuhause, dass es dann auch die Möglichkeit gibt, ehm einzustellen, dass man in nem entspannteren Umgangston miteinander redet. (EN:Yes, I think at home that there is then also the possibility, ehm to adjust that you talk to each other in a more relaxed tone. )* (Int. 13, pos. 156)

(25) *DE: Also ich würde erwarten, dass in der ersten Anfangszeit er natürlich unfassbar höflich ist, aber dass er darauf eingestellt ist, mich zu spiegeln in Anführungsstrichen, also meine Art und Weise zu kommunizieren, zu verstehen, zu verarbeiten und in seiner Art zu kommunizieren, mit aufzunehmen. Auch dass er dieses so ne krasse Höflichkeit natürlich fallen lässt, weil ich jemanden, der bei mir zu Hause sind, ja auch dauerhaft oder viel bei mir zu Hause ist, nicht als jemanden erwarte, der unfassbar höflich zu mir ist. Sondern jemand, der einfach in einem ganz normalen eine ganz normale Gesprächsart mit mir redet* *(EN:So I would expect that in the first initial period he is of course incredibly polite, but that he is set to mirror me in quotation marks, so to understand my way of communicating, to process and to include in his way of communicating. Also that he drops this extreme politeness of course, because I expect someone who is in my home, yes also permanently or a lot at my place, not to be someone who is incredibly polite to me. But someone who simply talks to me in a very normal a very normal way of conversation )* (Int. 8, pos. 110)

## 1.3 Visualization of themes and sub-themes

### 1.3.1 HHI Results

**Motivation**

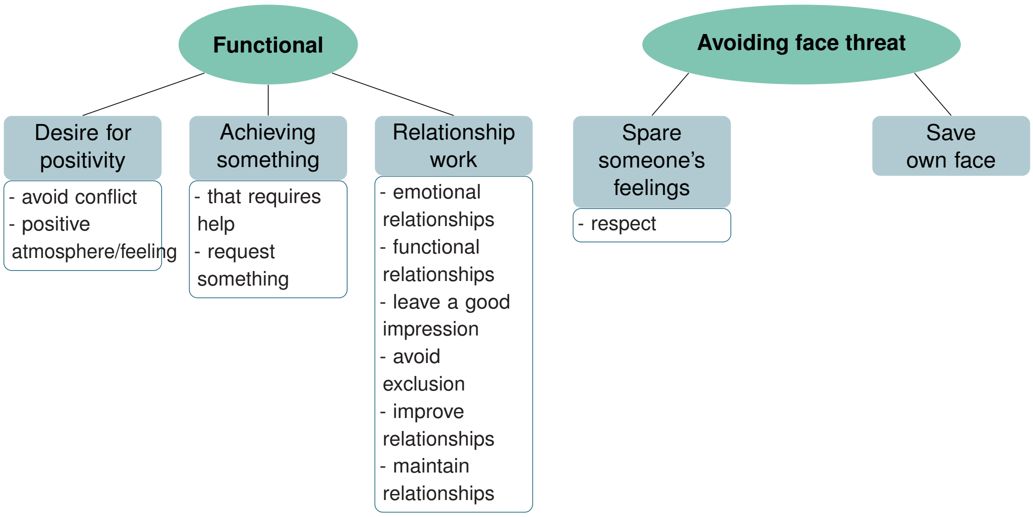


**Influences**

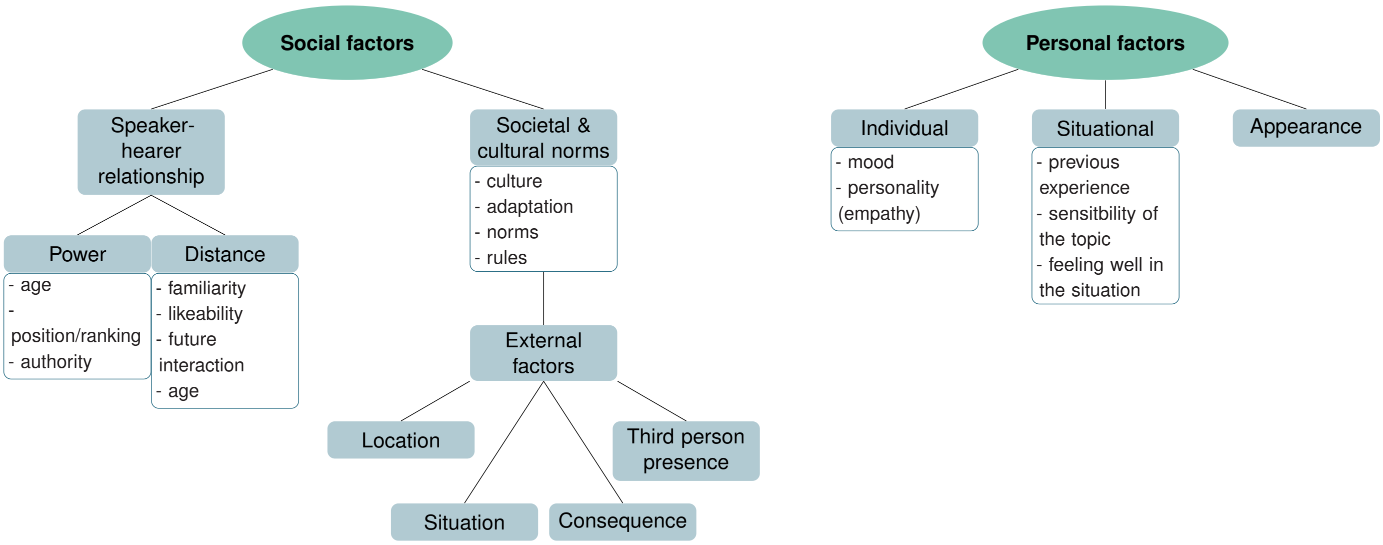


**Expectations for politeness strategies**

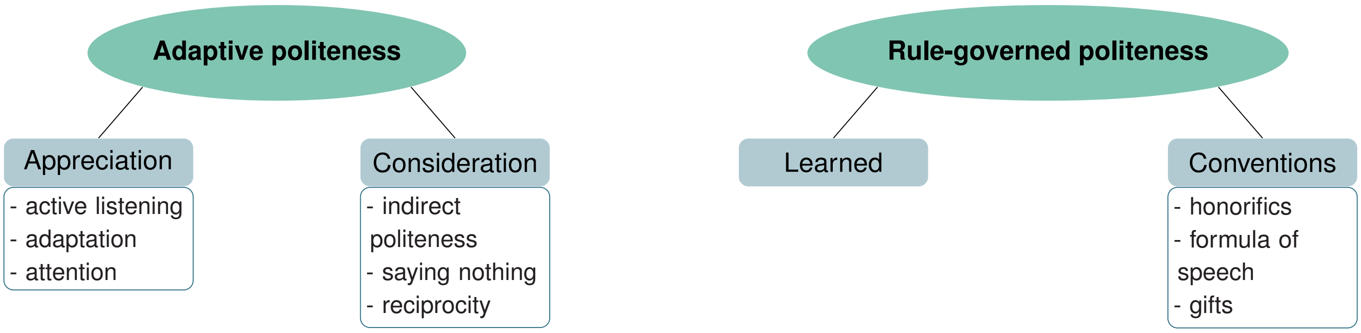

### 1.3.2 HRI Results

**Motivation**

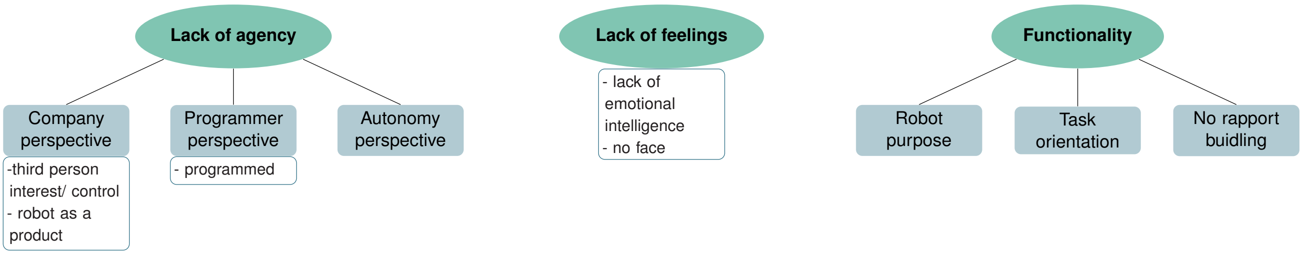


**Influences**

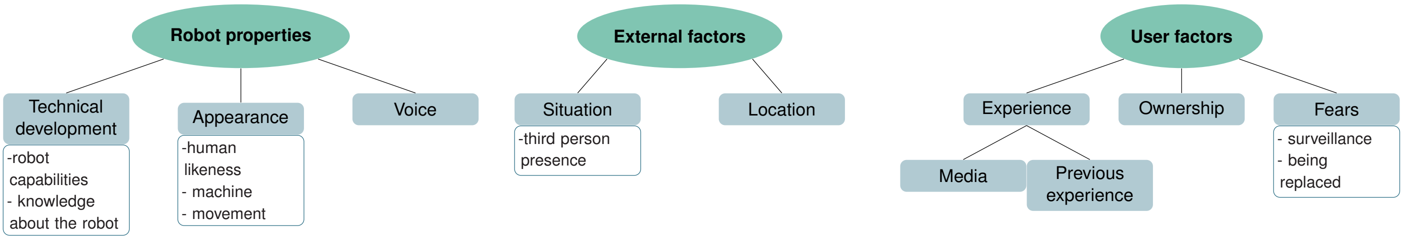


**Expectations for politeness strategies**

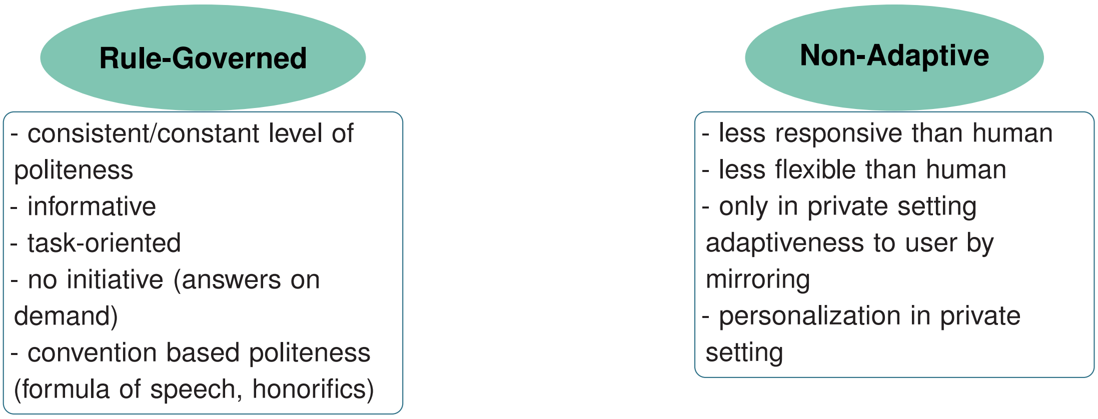

## 2 INTERVIEW GUIDE

This section presents the translation (from German) of the guide used for the interviews. The wording of questions and the order of questions, depending on the flow of conversation, could vary between participants, as the interviews were 'semi-structured'. Prior to the study, the interview guide was reviewed by an external researcher experienced with qualitative, interview-based research.

1. **Introduction and warm-up phase**
    - How was your arrival?
    - Short introduction by the moderator.
    - Short explanation of the interview procedure.
    - Signing of the consent form.
    - Starting the recording.
    - How did you learn about this study? (warm-up question)
2. **General Politeness**
   *(Goal: Query participant's understanding and perception of politeness)*
    - Politeness definition: What, in your opinion, is politeness?
        - Can you think of other types of politeness?
        - Sometimes there are situations where one is asked something that one does not want to answer directly/say the truth. Are you aware of such situations? If yes, how do you answer in these cases?
    - Example situation of politeness: Do you recall a situation in which you were polite to someone or someone was polite to you?
        - If no: Could you imagine such a situation?
        - If yes: Why did you perceive this as polite?
    - Has there ever been a situation in which someone was unexpectedly polite to you? (optional)
        - If no: Can you imagine such a situation?
        - If yes: How was this situation for you? And why was it unexpected?
3. **Influences on Politeness**
   *(Goal: To gain insights into the perceived influences on politeness.)*
    - Why, in your opinion, are people polite? What are possible reasons why people are polite to you?
        - In which situations can you imagine/do you expect that someone is polite? (follow-up)
    - Sometimes one may says something that a person wants to hear instead of what one actually thinks? Are you aware of such a situation?
        - Has this ever happened to you?
        - Do you have the impression that you are sometimes more polite to some people than to others?
        - What do you think influences your decision to be more polite to some people?
        - To whom do you think are you more polite and to whom more impolite or polite in different ways?
    - What kind of relationship do you need to have to someone, in order to find it inappropriate when they say, in a direct way, that you did something badly? (additional question)

4. **Revealing the Furhat robot**
   The interviewer moves a poster board to to reveal the Furhat robot. When revealed, the robot is in an active but idle state: The back-projected face is visible, its eyes may blink blinking from time to time, but it looks straight ahead into the room without physically moving. No 'skill' is running.
5. **General perception and attitude towards robots**
   *([Goal:] Introduction of robot and understanding of attitude towards robots in general and interaction with robots)*
   - Have you ever seen a robot like this?
   - Have you ever interacted with a robot? (including, e.g., cleaning robots)
     - Have you ever interacted with an voice assistant (e.g., Alexa, Siri, . . . )?
     - If yes: How was your experience? Have you noticed anything positive or negative when interacting with a voice assistant?
     - Would you expect this robot to talk in a similar way, as the voice assistant you interacted with?
   - Do you think that robots are always honest to you?
6. **Expectations regarding politeness-use by robots**
   *(Goal: Insights into the politeness expectations for robots compared to humans)*
   - Do you expect differences communication with a robot compared to a human?
     - What kind of differences?
     - Why do you (not) expect differences?
   - Imagine meeting this robot being set up on a small pedestal at a train station, acting as a service robot as you can see in this picture (see figure S1).
     - Do you expect that the robot speaks in similarly to a person working for the train company at the train station?
       - If no: What kind of differences do you expect?
       - If yes: How do you expect the interaction to be similar?
     - What would you expect the robot to say when someone does something wrong at the train station, e.g., dropping a chewing gum on the floor? Would you expect the robot to say something? If yes, what?
     - What would you expect the robot to say when something worse happens for instance when an act of vandalism happens by a person (e.g., destructing things at the station). Would you expect the robot to say something? If yes, what?
     - What would you expect from a person in this situation? Would you expect similar behavior/reaction by a human working there?
     - OFFICE: What about when the robot is in a different role in a different setting, e.g., when he is your assistant at work in the office? And, as before, someone drops a chewing gum on the floor?
     - HOME: What about when the robot is at your home? When it, for example, is in your living room and you can use it for entertainment purposes. If someone acts wrong, maybe not dropping a chewing gum on the floor, but something similar. Would you expect the robot to say something? If yes, what?
   - Smart home: Now imagine that the robot is connected to a smart home system. It can, for example, recognize when someone leaves the fridge open. You go from the kitchen to the living room, where

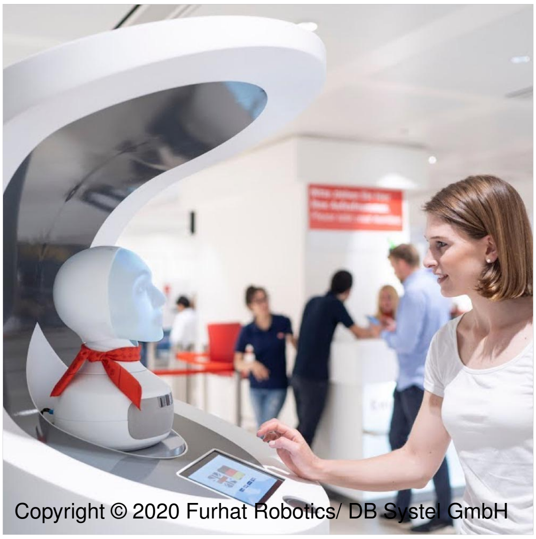


**Figure S1.** Picture of a woman at a train station interacting with a Furhat robot shown as an example to participants to help them imagine a scene with a robot in a public space.

the robot is, and it noticed that the fridge is still open. Would you expect it to say something? If yes, what?

  - Would you change this expectation when the situation is at your workplace/office and someone leaves the fridge open there?
  - Would you expect a human to say something similar in this situation, e.g., a friend at your place?

- You said that someone would be more polite to you if — (answer to previous questions in section 2 and 3) — you would expect that if you were _____ (relationship e.g. boss; trait e.g. big/nice) to a robot that they would then be also more polite to you?
  - Would you expect that when the robot is in your home, your relationship with the robot will be different?
  - As you said before, you talk to some people differently than to others (answer to question 5). If your relationship with a robot changes or you meet another robot that you have a different relationship with (for example because it is not at your home but at work/university), would you expect a different behavior?

7. **After interaction with the robot**
   - How did you like the interaction?
   - Would you now change your expectations you had earlier regarding the politeness behavior of robots?
8. **Debriefing**
   - Is there something that you would like to tell me that I haven not mentioned yet?
   - Do you have any questions in general or is there anything you would like to know?
   - Explanation of the research goal and purposes – if participant asks.
   - Questionnaire collecting demographic data.
   - Participant is given monetary compensation.
